# Differential Diagnoses of Overgrowth Syndromes: The Most Important Clinical and Radiological Disease Manifestations

**DOI:** 10.1155/2014/947451

**Published:** 2014-06-09

**Authors:** Letícia da Silva Lacerda, Úrsula David Alves, José Fernando Cardona Zanier, Dequitier Carvalho Machado, Gustavo Bittencourt Camilo, Agnaldo José Lopes

**Affiliations:** ^1^Department of Radiology, State University of Rio de Janeiro, 20551-030 Rio de Janeiro, RJ, Brazil; ^2^Postgraduate Programme in Medical Sciences, State University of Rio de Janeiro, 20550-170 Rio de Janeiro, RJ, Brazil

## Abstract

Overgrowth syndromes comprise a heterogeneous group of diseases that are characterized by excessive tissue development. Some of these syndromes may be associated with dysfunction in the receptor tyrosine kinase (RTK)/PI3K/AKT pathway, which results in an increased expression of the insulin receptor. In the current review, four overgrowth syndromes were characterized (Proteus syndrome, Klippel-Trenaunay-Weber syndrome, Madelung's disease, and neurofibromatosis type I) and illustrated using cases from our institution. Because these syndromes have overlapping clinical manifestations and have no established genetic tests for their diagnosis, radiological methods are important contributors to the diagnosis of many of these syndromes. The correlation of genetic discoveries and molecular pathways that may contribute to the phenotypic expression is also of interest, as this may lead to potential therapeutic interventions.

## 1. Introduction

Longitudinal growth results from multifactorial and complex processes that take place in the broader context of different genetic traits and environmental influences [1, 2]. Overgrowth syndromes comprise a heterogeneous group of disorders that lead to excessive tissue proliferation, which is characterized by a phenotype of excessive somatic and visceral growth [1–3]. A myriad of syndromes are characterized by substantial localized or asymmetric tissue overgrowth, represented by Beckwith-Wiedemann syndrome, Sotos syndrome, Proteus syndrome, Klippel-Trenaunay-Weber syndrome, Madelung's disease, neurofibromatosis type I, Weaver syndrome, Nevo syndrome, Simpson-Golabi-Behmel syndrome, Bannayan-Riley-Ruvalcaba syndrome, Perlman syndrome, Pallister-Killian syndrome, and many other conditions. The Beckwith-Wiedemann and Sotos syndromes are the most frequent [1–4]. Overgrowth syndromes can be localized or diffuse and often manifest at birth or in the postnatal period [4].

Though most growth syndrome have a genetic basis, others such as Madelung's disease have unknown etiology [4]. Genetic associations are already well established for some conditions including Weaver, Perlman, and Proteus syndromes [2]. The fact that Madelung's disease tends to occur in older males and is often associated with heavy alcohol consumption in 60–90% of cases suggests that it is an acquired abnormality or more susceptible to environmental modifiers [5, 6]. Importantly, most of these syndromes lead to increased risks of cognitive disorders and cancers [7]. The frequency of cancer is well documented in some syndromes such as Proteus (20%), Sotos (2–4%), and Perlman (65%) [2]. In some overgrowth syndromes, such as Beckwith-Wiedemann and Perlman syndromes, tumors appeared mostly in the abdomen; conversely, in other overgrowth syndromes, such as Sotos syndrome, the most frequent type of overgrowths is extra-abdominally located lymphohematological tumors [8].

A dysfunction in the receptor tyrosine kinase (RTK)/PI3K/AKT pathway that specifically promotes a rapid increase in insulin receptor expression is most likely the pathophysiological basis of some overgrowth syndromes [4]. Insulin receptor stimulation leads to an increase in PI3-kinase activity, which thereby generates PIP3 and leads to the activation of PKB/Akt [4]. This pathway plays an important role in the activation of somatic mutations in various tumors as well as in apoptosis, angiogenesis, and brain development [9–11]. A dysfunction in the (RTK)/PI3K/AKT pathway has been described in some conditions such as Proteus syndrome [4]. The genetic factor is intrinsically involved in some overgrowth syndromes. It is noteworthy to highlight the imprinted growth regulatory genes on chromosome 11p15.5. In this region, there is one domain in which the* H19* expression from the maternal allele is thought to protect against the tall stature. There is also a second domain that consists of the* CDKN1C* gene, which acts as an in-utero negative regulator of cell growth. Heritable forms of Beckwith-Wiedemann syndrome have been attributed mainly to mutations in the growth suppressor gene* CDKN1C* [2, 12]. There are some reports of* NSD-1* mutations in individuals with Sotos and Weaver syndromes and more recently mutations in the* EZH2* gene in three families with Weaver syndrome have been identified [13]. A previously unknown susceptibility locus was mapped and germline mutations in* DIS3L2* identified in individuals with Perlman syndrome. Functional studies demonstrated that underexpression of the* DIS3L2 *gene was associated with cellular growth enhancement [14].

Several classifications have been developed in an attempt to facilitate the diagnosis of these syndromes, but these attempts have been hindered by the syndromes' several overlapping clinical manifestations [1, 15]. Neylon et al. [2] proposed a classification of overgrowth syndromes by ordering them according to their typical timing of clinical presentation as follows: (a) syndromes exhibiting overgrowth in the neonatal period, including Beckwith-Wiedemann syndrome, Sotos syndrome, Weaver syndrome, and Perlman syndrome and (b) overgrowth syndromes usually identified in childhood, including Klinefelter syndrome and Proteus syndrome. Major progress such as the identification of genetic causes has recently enhanced the knowledge of the underlying pathophysiological mechanisms, the delineation of the genotype-phenotype relationships, and the establishment of the main characteristics for each condition [1]. As a consequence, the possibilities for distinguishing between different overgrowth syndromes have increased. Several studies are currently underway to organize these types of disorders according to a molecular classification system for overgrowth syndromes in order to assist the practicing clinician [16–18]. Radiological abnormalities are increasingly important for the clinical differentiation between overgrowth syndromes, making those abnormalities valuable diagnostic criteria for some of these conditions.

In this review, four overgrowth syndromes—Proteus syndrome, Klippel-Trenaunay-Weber syndrome, Madelung's disease, and neurofibromatosis type I—are described. The main clinical and imaging features these syndromes are highlighted using clinical cases evaluated in our institution. Although they are not the most common overgrowth syndromes, manifestations of these four syndromes overlap with other more prevalent overgrowth syndromes. Thus, it is of interest to present these cases which were diagnosed from the suspicion caused by imaging findings.

## 2. Proteus Syndrome

Proteus syndrome is a congenital disorder of unknown etiology, and it is the prototype of overgrowth syndromes. It was first described in 1979 and is characterized by multisystem involvement and clinical variability [19]. This disorder became prominent in 1980 after being depicted in the movie* The Elephant Man* [19, 20]. Proteus syndrome is a rare condition with an estimated prevalence of one in 1 million people worldwide [21]. A study showed a somatic activating mutation of the* AKT1* oncogene kinase, an enzyme involved in cell proliferation, in this disorder [22]. This finding implies the activation of the* PI3K-AKT* pathway in the characteristic clinical findings of overgrowth and tumor susceptibility in patients with Proteus syndrome [22].

Proteus syndrome can affect all three germ lineages. Abnormal asymmetric growth and hemihypertrophy are its typical clinical manifestations, though overgrowth of the long bones, macrodactyly, asymmetric macrocephaly, plantar or palmar hyperplasia, vertebral abnormalities, lipoma, hemangioma, connective tissue nevi, lymphangiomas, and vascular malformations can also be observed in this syndrome [7, 23–25]. Because there is no specific genetic testing, the diagnosis of this syndrome is based on clinical data and radiological evolutions according to the criteria formulated in 1998 by the National Institutes of Health [19, 23]. The primary hallmark of Proteus syndrome is a mosaic or random distribution of lesions throughout the body that develop gradually during childhood, after which point the disease can stabilize or continue to slowly progress [23]. Some authors believe that the disease becomes stable at approximately 15–17 years of age [21, 26].

**Figure 1:**
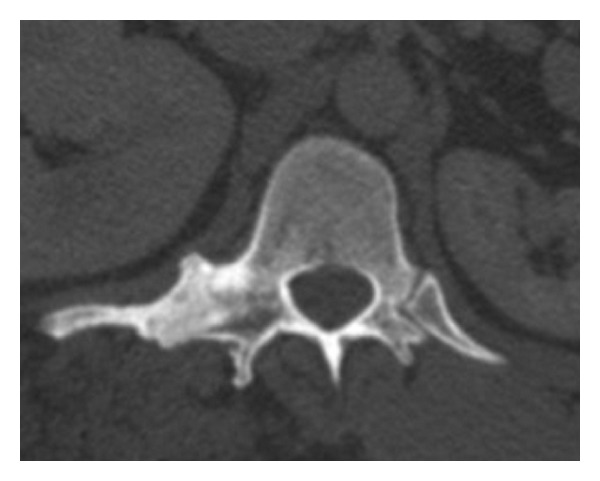
The right costovertebral joint space is fused. The T12 vertebra shows disproportionate asymmetric overgrowth which is characteristic for the Proteus syndrome.

**Figure 2:**
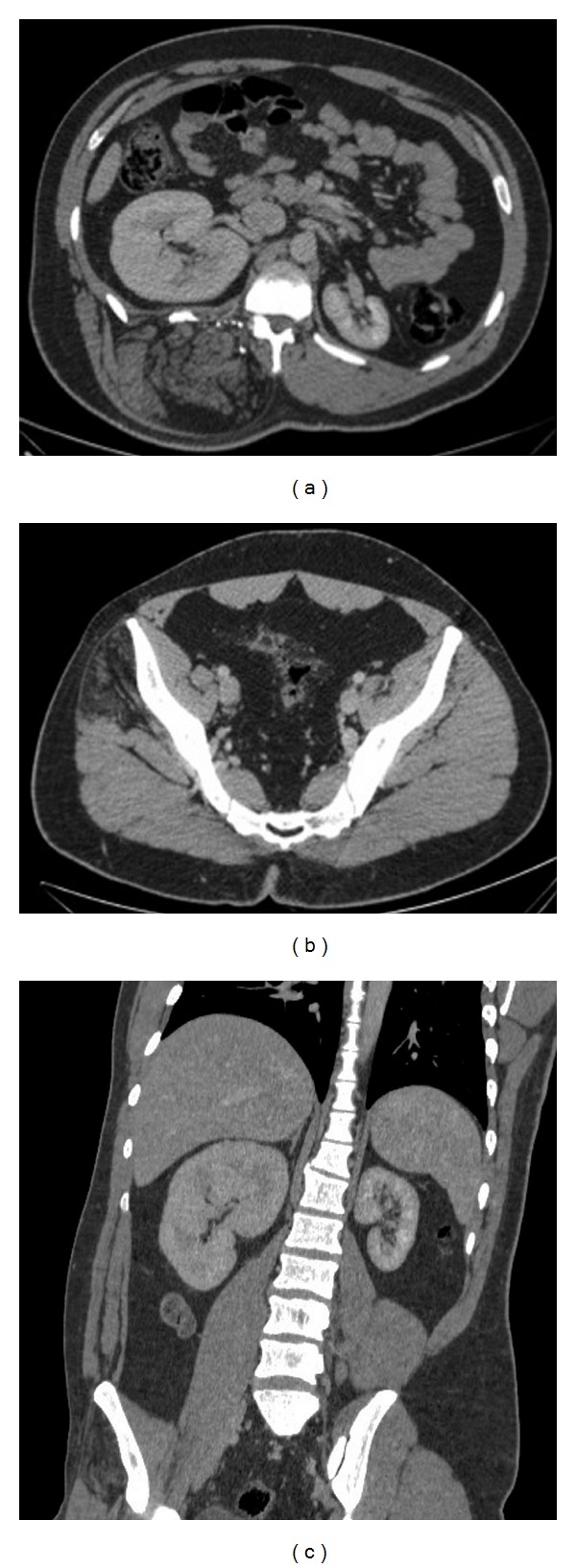
A 21-year-old man with Proteus syndrome presenting asymmetric lower limbs and epidermal nevus. CT of the abdomen showed tissue with a fat density infiltrating the right paraspinal musculature, with increased local volume extending from T7 to L5, in addition to an enlargement of the right kidney (a). The scan also noted fatty replacement in right gluteal muscles (b). Coronal CT showed asymmetry of the kidneys (c).

Skeletal changes are the most frequently expressed manifestations of Proteus syndrome and include kyphoscoliosis, macrodactyly, hyperostosis, asymmetric overgrowth of limbs, abnormal vertebral bodies (Figure 1), craniofacial abnormalities, and focal calvarial thickening [19, 23, 25]. Among the soft tissue manifestations, asymmetric growth of the subcutaneous tissue (Figure 2) is common and may be associated with exacerbated muscle development and the proliferation of lymphatic channels and vascular malformations [19, 23]. Connective tissue nevi may also be observed, particularly in the plantar region, as well as cerebriform nevi [23]. Cerebral arteriovenous malformations, abnormal grey-white matter differentiation, and hydrocephalus are also common findings. Visceral changes, such as splenomegaly or nephromegaly (Figure 2), hydronephrosis, pancreatic lipomas, colonic polyps, emphysema, and lung cysts are less common findings [19, 23, 24]. All of these conditions aid in the differential diagnosis of Proteus Syndrome, which can be challenging because Klippel-Trenaunay-Weber syndrome, Maffucci's syndrome, enchondromatosis, neurofibromatosis type I, Bannayan-Zonana syndrome, hemihyperplasia, and Madelung's disease can also cause overgrowth [24, 27]. Importantly, disproportionate asymmetric overgrowth can be a clue to the differential diagnosis of other diseases of osseous overgrowth in which the enlarged bones retain their normal proportional relationships [24–26].

## 3. Klippel-Trenaunay-Weber Syndrome

Klippel-Trenaunay-Weber syndrome is rare and has an uncertain origin with an incidence of approximately 1 : 100,000 live births [28]. It appears to have no predilection for gender or race, and most of the cases are sporadic and appear at birth [29, 30]. The French physicians Maurice Klippel and Paul Trenaunay first described this syndrome in 1900 when they associated vascular malformations with hypertrophy in the affected limb. Subsequently, arteriovenous fistulas in these patients were described by Parkes Weber [30–32]. Several theories attempt to elucidate the etiology of this syndrome, such as multifactorial, paradominant inheritance, or mosaic mutation [33]. Some authors state that a deep venous obstruction or atresia can lead to swelling and limb hypertrophy [30]. Others state that the disease symptoms are caused by a change in the angiopoietin-2 antagonist, which determines the maintenance of small arteriovenous communications in the limbs [34]. However, further experts argue that the hypertrophy observed in soft tissues is a primary occurrence that occurs independently of fistulas [35].

**Figure 3:**
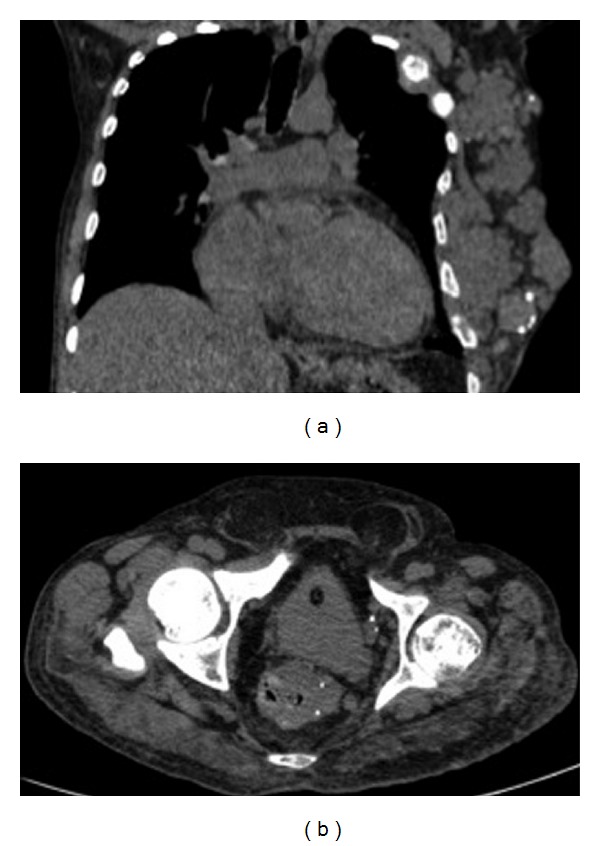
A 60-year-old man with Klippel-Trenaunay-Weber syndrome presenting asymmetric growth of the lower limbs. CT of the chest showed increased soft tissue as well as extensive vascular malformations in the left hemithorax wall with intermingled phleboliths, causing multiple lytic lesions with enlargement in the ipsilateral ribs (a). CT of the abdomen showed a thick-walled rectum intermingled with phleboliths, denoting varicose veins (b).

Klippel-Trenaunay-Weber syndrome is characterized by the presence of capillary malformations associated with venous malformations or varicose veins (Figure 3) and with bone or tissue hypertrophy; a diagnosis of this syndrome is based on the presence of at least two of these three categories [29, 36]. 63% of diagnosed patients present all three symptoms [35]. The most common manifestation, present in 98% of patients, is capillary malformation, which is represented by cutaneous hemangiomas or a port-wine stain [29, 36]. These lesions usually affect the hypertrophied limb, and when they occur in the trunk region, they rarely cross the midline [29, 37–39]. Varicose veins are also present in most patients with Klippel-Trenaunay-Weber syndrome, and they are more evident during adolescence and affect both the superficial and deep venous systems [26, 29]. The varicose veins may remain stable or progress, causing pain, lymphedema, thrombophlebitis, and ulcers [30]. Hypertrophy, usually resulting from venous ectasia, is always secondary to issues involving bone, soft tissue, or both, which distinguishes this syndrome from Proteus syndrome, in which bone and tissue overgrowth can occur independently of vascular malformations [23, 30, 35, 40].

Other features also differentiate these two syndromes. Klippel-Trenaunay-Weber syndrome is bilateral and less frequently involves the upper limbs [23]. Some authors believe that in Proteus syndrome, the limb overgrowth is usually mild or absent at birth, while in Klippel-Trenaunay-Weber syndrome it is present and severe at birth. Other authors have stated that limb hypertrophy is the latest indicator of Klippel-Trenaunay-Weber syndrome [30]. Bone overgrowth, which is dysplastic, progressive, and irregular, is typical of Proteus syndrome and not observed in Klippel-Trenaunay-Weber syndrome; thus, its detection is an important tool in differentiating between the diseases [23].

## 4. Madelung's Disease

Madelung's disease is also known as multiple symmetric lipomatosis, benign symmetric lipomatosis, or Launois-Bensaude adenolipomatosis. It is a rare condition that is possibly related to alcohol consumption and leads to denervation and subsequent adipocyte hypertrophy [41, 42]. Alcohol appears to play a role in the adipocyte hyperplasia process in genetically susceptible individuals through the prolipogenesis and antilipolytic effects [43]. However, other studies have also suggested the presence of mitochondrial inheritance through mutation of the maternal gene [44, 45]. Madelung's disease is most common in adult males of Mediterranean descent [46], with an estimated incidence of 1 : 25,000 in Italy [47].

It is manifested by the painless deposition of multiple nonencapsulated masses of fatty tissue, which are symmetrically distributed in the cervical and upper thoracic regions over a period of months to years. The face, hands, and feet are usually unaffected. A Madelung's disease diagnosis is based on an ectoscopy as well as additional tests that rule out the skin, vascular, and bone changes present in other diseases [48]. This disease often leads to aesthetic complaints, but it is rarely associated with complications such as dyspnea (caused by upper airway compression) or dysphonia (caused by an involvement of the recurrent laryngeal nerve). Madelung's disease is classified as type I when lipomatous masses are observed in the parotid, cervical, suprascapular, or deltoid regions and classified as type II when the lipomatosis is diffuse, resembling simple obesity [48].

**Figure 4:**
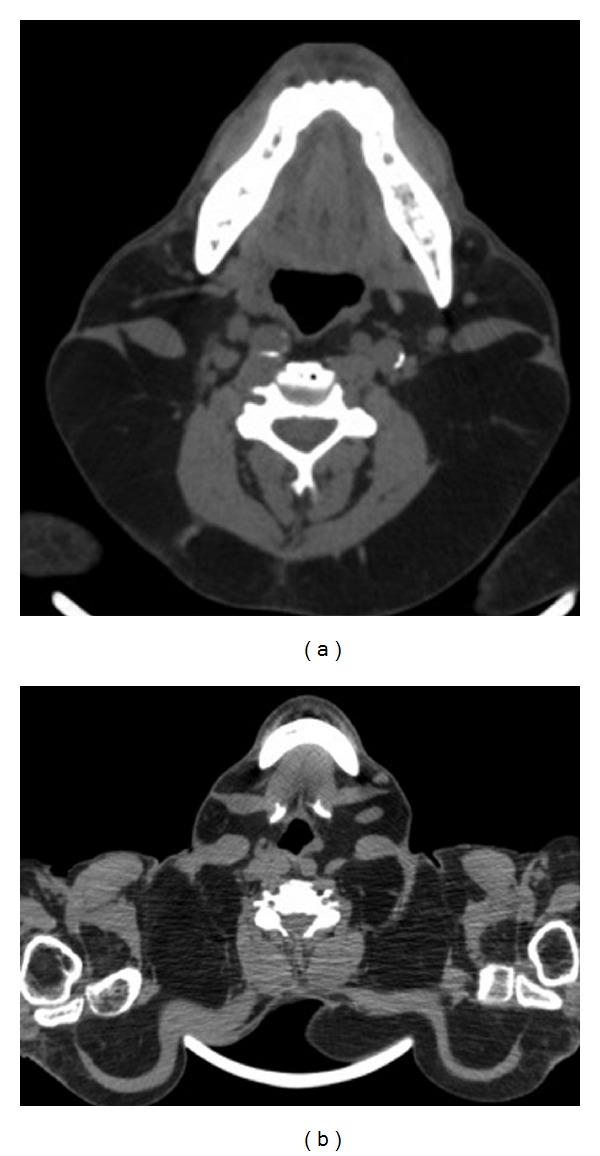
A 53-year-old man with Madelung's disease presenting a progressive painless increase of the cervical region. CT of the neck and chest showed fat deposition occurring predominantly in the posterior subcutaneous region of the neck (a) and in the supraclavicular and upper regions of the chest (b).

Computed tomography (CT) is important for a Madelung's diagnosis because it can identify the key symptoms, such as lipomatosis in the characteristic regions (Figure 4), the calcification of lipomas, tracheal narrowing, and venous stasis in the chest wall, while confirming the absence of masses in other sites [46]. When performing a differential diagnosis, diseases in addition to other overgrowth syndromes must be considered. When there are similar cases in the family, familial lipomatosis is an option, and Dercum's disease (adiposis dolorosa) is a possibility if the fat accumulation is accompanied by pain [23].

## 5. Neurofibromatosis Type I

Neurofibromatosis type I, also known as von Recklinghausen's disease, was first described in 1882 by Friedrich Daniel von Recklinghausen. Neurofibromatosis type I is the most common type of phakomatosis or neurocutaneous syndrome, occurring in one out of every 2000 live births with no predilection for gender or race [49, 50]. It is an autosomal dominant disorder caused by heterozygous mutations of the NF-1 gene, located at chromosome 17q11.2 [51]. The NF1 gene encodes a large cytoplasmic protein called neurofibromin, which is a major negative regulator of* Ras* protooncogene, a key protein in a major signal transduction pathway [50, 52]. In half of the cases, however, this disease occurs sporadically via spontaneous mutations that cause abnormal growth in nervous and fibrous tissues [49, 50].

Clinical symptoms are usually observed in childhood, though in approximately 10% of cases they occur later in life and are atypical [48, 53]. Neurofibromatosis type I can exhibit different clinical manifestations, which makes a diagnosis more difficult. Generally, the disease affects the skin, nervous system, bones, and endocrine glands by causing benign tumors [49]. The diagnostic criteria for this disease were developed in 1987 and redefined in 1997 [50], and they are based on the presence of two or more of the following findings: a first-degree relative who has neurofibromatosis type I, “café-au-lait” spots, neurofibromas, freckles in the axillary or inguinal regions, optic gliomas, iris hamartomas, and distinctive bone lesions.

**Figure 5:**
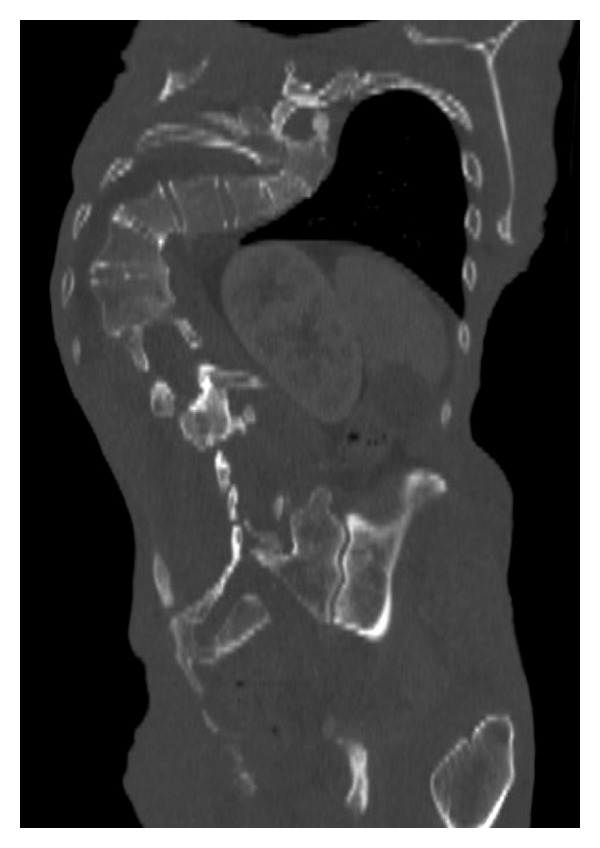
A 53-year-old man with neurofibromatosis type I. A morphostructural abnormality in the spine is characterized by significant dorsolumbar scoliosis with right convexity, as observed in his CT scan (coronal section).

The “café-au-lait” spots are present in approximately 95% of diseased patients and are usually congenital; they occur in different sizes and are distributed throughout the body surface [50, 54]. Among the most frequent skeletal abnormalities observed in neurofibromatosis type I are scoliosis (Figure 5), kyphosis, growth disorders, pseudarthrosis of long bones, and sphenoid wing dysplasia [55]. Over time, patients with neurofibromatosis type I may experience abnormalities of the skeleton (thinning or overgrowth of the bones in the arms or lower leg) [50, 55].

**Figure 6:**
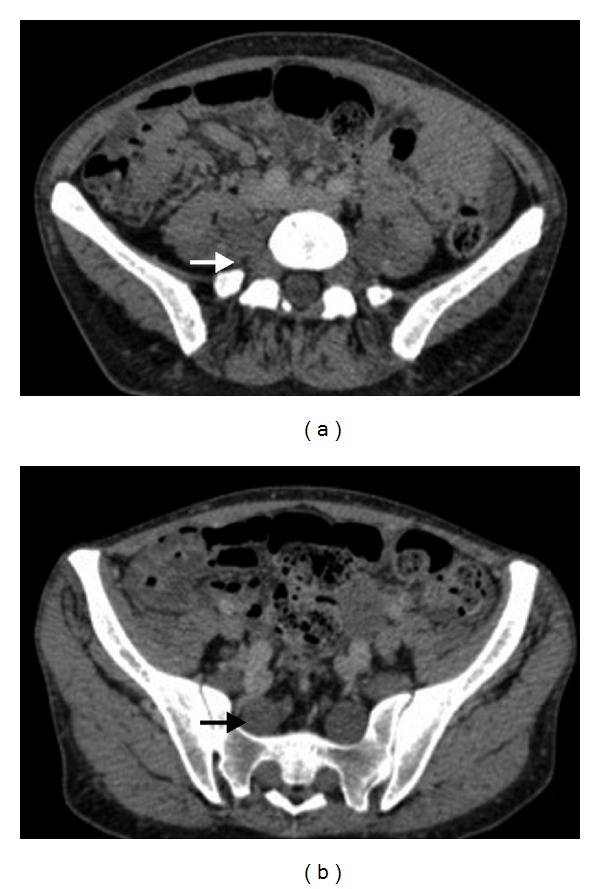
A 34-year-old woman with neurofibromatosis type I. Axial CT show plexiform neurofibromas of lumbar and sacral nerve roots.

Neurofibromas are the tumors of the peripheral nervous system typically observed in this disease, particularly plexiform neurofibromas [49], which are derived from Schwann cells and fibroblasts. Approximately 30% of patients with a single neurofibroma will develop neurofibromatosis type I, and virtually all patients with multiple neurofibromas, especially of the plexiform type (Figure 6), have the disease [49]. Iris hamartomas (Lisch nodules) are bilateral and asymptomatic hamartomatous lesions on the surface of the iris. Multiple hamartomas are unique to neurofibromatosis type I.

Several of the diagnostic criteria are confirmed by radiological examinations. Tomographic findings depend on the histological features of the tumors and may exhibit soft tissue density. More commonly (in 73% of cases) there is low attenuation due to cystic degeneration, confluent areas of hypocellularity, or lipid abundance [49]. Neurofibromatosis is distinguished by its typical symptoms including neurofibromas, Lisch nodules, axillary freckles, and “café-au-lait” spots, which are absent in other overgrowth syndromes [23].

## 6. Conclusion

Overgrowth syndromes are characterized by diffuse or localized tissue proliferation and they may originate in a dysfunctional receptor tyrosine kinase (RTK)/PI3K/AKT pathway. These syndromes represent a heterogeneous group of diseases with manifestations that often overlap each other, requiring the use of preestablished diagnostic criteria in most cases.

In this review, four overgrowth syndromes were characterized according to their primary clinical and radiological features. Identifying these features is important for making the correct diagnosis and to appropriately monitor the patient's health because no specific genetic tests for these syndromes are available.
